# Denture-related problems of patients in acute geriatric care

**DOI:** 10.1007/s00391-021-01928-1

**Published:** 2021-06-25

**Authors:** Ina Nitschke, Frederick Frank, Ursula Müller-Werdan, Rahel Eckardt-Felmberg, Angela Stillhart

**Affiliations:** 1 Clinic of General, Special Care and Geriatric Dentistry, Center of Dental Medicine, University of Zurich, Zurich, Switzerland; 2 Division of Gerodontology, Clinic of Prosthetic Dentistry and Dental Materials Science, Leipzig University Medical Center, Leipzig, Germany; 3 Department of Geriatrics and Medical Gerontology, Charité—Universitätsmedizin Berlin, and Protestant Geriatric Center Berlin, Berlin, Germany; 4 St. Joseph Hospital, Berlin, Germany

**Keywords:** Geriatric dentistry, Acute geriatric hospital, Teeth, Dentures, Oral treatment needs, Seniorenzahnmedizin, Akutgeriatrie, Zähne, Zahnersatz, Dentaler Behandlungsbedarf

## Abstract

**Background:**

With increasing frailty and complaint-oriented utilization of dental care, the prevalence of oral diseases also increases.

**Aim:**

To clarify whether there is a need for dental prosthodontic treatment during residential acute geriatric rehabilitation.

**Methods:**

Within 3 weeks in a hospital for acute geriatric patients, 79 out of 157 newly admitted patients were interviewed as study participants (age: median 79.0 years, range 66–96 years, female 51.9%), dental findings were recorded, treatment needs were determined but X‑rays were not taken.

**Results:**

Of the participants 31.1% had not seen a dentist for more than 1 year and 18.2% were edentulous. The median number of teeth in dentate participants was 16 (range 1–28 teeth); based on all participants, there was a median of 12.0 teeth (range 0–28 teeth). Of the 52 denture wearers (45 upper jaw and 43 lower jaw), 5 each of the maxillary and mandibular dentures could not be assessed because they were not available at the hospital. Moderate denture deficiencies were present in 62.5% of participants wearing upper dentures (mandibular 55.3%).

**Conclusion:**

Dental treatment is needed in this vulnerable patient group. Therefore, the oral cavity should be assessed as part of the geriatric assessment. The available data confirm that the use of validated assessment instruments, such as the mini dental assessment as part of the comprehensive geriatric assessment would be useful. In addition to an oral examination, simple dental treatment should be provided to reduce infections and improve chewing ability. The geriatrician should be informed of the urgency of treatment. The overall rehabilitative approach of acute geriatric treatment would be complete if oral health would not be excluded.

## Abstract (de)

**Hintergrund:**

Mit zunehmender Gebrechlichkeit und beschwerdeorientierter Inanspruchnahme des Zahnarztes steigt auch die Prävalenz von Erkrankungen der Mundhöhle.

**Ziel:**

Es soll geklärt werden, ob ein zahnärztlich-prothetischer Behandlungsbedarf während einer stationären akutgeriatrischen Rehabilitation besteht.

**Methoden:**

Innerhalb von 3 Wochen wurden in einem Krankenhaus für Akutgeriatrie von 157 neuaufgenommen Patienten 79 als Studienteilnehmende (Altersmedian: 79,0 Jahre, Range: 66–96 Jahre, davon Frauen: 51,9 %) strukturiert interviewt, ein zahnärztlicher Befund aufgenommen und der Behandlungsbedarf dann abgeleitet. Röntgenbilder wurden nicht erstellt.

**Ergebnisse:**

Von den Studienteilnehmenden waren 31,1 % länger als ein Jahr nicht beim Zahnarzt, 18,2 % waren zahnlos. Die mittlere Zahnzahl der bezahnten Studienteilnehmenden betrug 16 Zähne (Range: 1–28), bezogen auf alle Teilnehmenden waren im Mittel 12 Zähne (Range: 0–28) vorhanden. Von den 52 Prothesenträgern (45 im Oberkiefer; 43 im Unterkiefer) konnten jeweils 5 Prothesen des Ober- und Unterkiefers nicht beurteilt werden, da sie im Krankenhaus fehlten. Bei 62,5 % der Studienteilnehmenden, die im Oberkiefer eine Prothese tragen (Unterkiefer: 55,3 %) lagen mäßige Mängel am Zahnersatz vor.

**Schlussfolgerung:**

Bei dieser vulnerablen Patientengruppe besteht zahnärztlich-prothetischer Behandlungsbedarf. Die Mundhöhle sollte daher im Rahmen des Geriatrischen Assessments befundet werden. Die vorliegenden Daten bestätigen, dass der Einsatz validierter Assessment-Instrumente, wie z. B. das Mini-Dental-Assessment als Teil des Comprehensive Geriatric Assessment sinnvoll wäre. Aus der Erhebung des oralen Befundes können sich während der geriatrischen Behandlung einfache zahnärztliche Behandlungen zur Reduktion von Infektionen und zur Verbesserung der Kaufähigkeit anschließen. Der Geriater sollte zur Therapiedringlichkeit informiert werden. Der gesamtrehabilitative Ansatz einer akutgeriatrischen Therapie wäre vervollständigt, wenn die Gesundheit der Mundhöhle nicht ausgeschlossen würde.

Oral health and multimorbidity are not contradictions if all stakeholders promote dental care; however, the reality of care shows that there are deficits in the oral health of geriatric patients. The reasons are multifactorial; with increasing frailty access to dental care becomes more difficult. During inpatient acute geriatric treatment the question arises whether the examination of the oral cavity should also be integrated into the geriatric complex treatment.

## Background

The proportion of seniors with their own teeth and dentures has increased [22, 23]. The fifth German Oral Health Study (DMS V) [7] illustrates dental prevention successes among seniors. Due to the decrease in tooth loss, the proportion of edentulous younger seniors (65–74 years) has halved since 1997 [17] from 24.8%, 22.6% in 2005 [9], to 12.4% in 2014 [23]. Of the very old 32% (75–100 years old) are edentulous [22] and 52% of people depending on care are edentulous [21]. In contrast to non-care-dependent seniors, seniors with care needs have poorer oral health [1, 8, 21]. The mean number of missing teeth among 65–74-year-olds was 17.6 teeth in 1997 [17], 14.2 teeth in 2005 [9] and 11.1 teeth in 2014 [23] (based on 28 teeth).

As frailty increases, visits to the dentist become less control-oriented [10, 24]. In the case of ambulatory care dependency, equal access to dental care is less likely to be available [4]. Oral health is no longer a priority [26], which increases the need for dental treatment in the long term. This raises the question of whether it would make sense to integrate a dental care service during geriatric complex treatment. Good oral health without periodontally induced infections with suitable dental prostheses contributes to the prevention of general medical diseases. The article aims to address the question whether there is a need for dental treatment, using the example of removable dentures, and whether a dental examination and treatment offer should be integrated into acute geriatric complex rehabilitation.

## Study design and research methods

The study was conducted at the Protestant Geriatric Center Berlin with 152 acute inpatient hospital beds. Data for the qualitative observational study were collected during the study period from 13–30 January 2016. The ethics committee of the Berlin Charité approved the implementation of the study (ethics vote of 25 June 2015, application number EA 1/139/15).

### Inclusion and exclusion criteria

Patients admitted to hospital who had given written, revocable consent to participate in the study (informed consent), either independently or through their legal guardian, were included. Exclusion criteria were a lack of consent or a terminal palliative condition as per the assessment of the treating geriatricians.

### Study procedure

Dentally relevant information regarding participants’ medical history, diagnoses and medication was obtained from the treatment management software NEXUS.MedFolio® [18] in compliance with all data protection regulations. A structured interview with 51 questions was conducted and oral findings were collected. For participants who were unable to attend due to health or organizational reasons, the interview and findings were conducted separately. Two participants could not attend the clinical examination after the interview due to illness-related reasons. Differences in the numbers of participants in the individual evaluation characteristics are due, among other things, to the fact that some participants could not or did not want to answer all the questions.

### Clinical examination

The dental examination in a well-illuminated oral cavity by means of a dental mirror, probe and a periodontal probe was carried out according to the guidelines of the DMS V [7]. In some cases, dentures could only be assessed outside the mouth (e.g. type of denture, visible defects) because the participant did not want to put the dentures into the mouth for example due to pain in cases of ill-fitting dentures.

### Oral functional capacity

Oral functional capacity (OFC), a gerostomatological assessment element, was determined with three parameters: therapeutic capability, oral hygiene ability, self-responsibility. The parameter with the lowest value is used to classify the participants into one of the resilience capacity levels (RCL 1–RCL 4) (Table 1; [19]).

**Table 1:** Description of the oral functional capacity (OFC) consisting of four resilience capacity levels (RCL 1–RCL 4) and three parameters (therapeutic capability, oral hygiene ability, self-responsibility). The parameter with the lowest value is used to classify the patients into one of the RCLs [19]

Resilience capacity level (RCL)	Therapeutic capability	Oral hygiene ability	Self-responsibility
RCL 1 normal	Normal	Normal	Normal
RCL 2 slightly reduced	Slightly reduced	Slightly reduced	
RCL 3 greatly reduced	Greatly reduced	Greatly reduced	Reduced
RCL 4 no resilience	None	None	None

### Quality of dentures and prosthetic treatment need

The need for treatment was determined by assigning the quality of the prostheses to one of four levels: very good, good, moderate or poor. Dentures were rated as moderate if they could be functionally restored with repair, relining or extension and as poor if a new fabrication was required [15]. In the case of very good or good dentures there was no or very little chairside need for treatment.

### Statistics

The data were processed within the framework of descriptive statistics using relative frequency tables. The statistical analysis was carried out with the software IBM Corp. Released 2017. SPSS Statistics for Windows, version 25 (IBM Corp., Armonk, NY, USA).

## Results

A total of 79 participants (median: 79.0 years, range: 66–96 years, women: 51.9%, 50.3% of all newly admitted 157 inpatients during this period) were interviewed and dental findings were recorded for 77 of these 79 participants.

### Utilization of dental services

Of the participants 31.6% had not seen a dentist for more than 1 year (Table 2) and 54.4% mentioned a check-up of the oral cavity as the reason for their last visit to the dentist.

**Table 2:** Time span since the last dental visit depending on the resilience capacity levels within the oral functional capacity (*n* = 74)

Time span	Resilience capacity levels								All	
	Normal		Slightly reduced		Greatly reduced		None			
	*n*	%	*n*	%	*n*	%	*n*	%	*n*	%
≤ 1 year	5	83.3	19	76.0	21	63.6	6	60.0	51	68.9
≤ 2 years	1	16.7	3	12.0	1	3.0	2	20.0	7	9.5
≤ 5 years	0	0	1	4.0	5	15.2	2	20.0	8	10.8
> 5 years	0	0	2	8.0	6	18.2	0	0	8	10.8
*Total*	6	100	25	100	33	100	10	100	74	100

### Number of teeth and dentures

Of the 77 participants 18.2% were edentulous (men: 9.1%, women: 9.1%, maxilla: 35.1%, mandible: 20.7%). The median number of teeth in dentate participants (*n* = 63) was 16 teeth (range: 1–28 teeth, mean ± SD: 15.3 ± 8.3 teeth); based on all participants, there was a median of 12.5 teeth (range: 0–28 teeth, mean ± SD: 12.5 ± 9.5 teeth). Of the missing teeth 76.1% were replaced with artificial teeth (based on 28 teeth). Of the missing 15.5 teeth 10.6 teeth (68.4%) were replaced by removable prostheses (dentures with artificial teeth or implant-supported removable prosthesis with artificial teeth) (Table 3).

**Table 3:** Replacement of missing teeth by bridge pontics with fixed dentures, by prefabricated replacement teeth in a removable prosthesis or by placing oral implants. Values for the German population from the fifth German Oral Health Study (DMS V), study group younger seniors 65–74 years [23] and older seniors 75–100 years [22]. The calculation is based on 28 teeth

	66–95 years			Young seniors 65–74 years						Old seniors 75–100 years					
	All study participants			Study participants			German population			Study participants			German population		
	All	Female	Male	All	Female	Male	All	Female	Male	All	Female	Male	All	Female	Male
	*n* = 77	*n* = 39	*n* = 38	*n* = 18	*n* = 10	*n* = 8	*n* = 1042	*n* = 553	*n* = 489	*n* = 59	*n* = 29	*n* = 30	*n* = 1133	*n* = 686	*n* = 447
	Ø	Ø	Ø	Ø	Ø	Ø	Ø	Ø	Ø	Ø	Ø	Ø	Ø	Ø	Ø
Missing teeth	15.5	15.4	15.7	10.5	12.0	8.7	11.1	11.2	11.0	17.0	16.5	17.5	17.8	18.5	16.8
Pontics	1.0	1.1	0.9	1.9	1.8	2.0	1.6	1.7	1.5	0.7	0.8	0.6	1.1	1.0	1.2
Removable prostheses	10.6	10.7	10.4	2.8	4.4	0.8	7.5	7.8	7.2	12.9	12.9	13.0	14.8	15.6	13.6
Implants	0.22	0.2	0.25	0	0	0	0.22	0.18	0.26	0.29	0.39	0.27	0.27	0.25	0.31
Total replacements	11.8	12.0	11.6	4.7	6.2	2.8	9.3	9.7	9.0	13.9	14.1	13.9	16.2	16.9	15.1
Unrestored tooth gaps	3.7	3.4	4.1	5.8	5.8	5.9	1.8	1.5	2.0	3.1	2.4	3.6	1.6	1.6	1.7
Missing teeth replaced (%)	76.1	77.9	73.9	44.8	51.7	32.2	83.8	86.6	81.8	81.8	85.5	79.4	91.0	91.1	89.9

### Wearing habits and satisfaction

In the upper jaw 45 patients had a denture, 5 patients did not bring their dentures to hospital (*n* = 40) (dentures in the lower jaw: 43 patients, 5 patients did not bring their dentures to hospital, *n* = 38). More than 80% of the participants wore their removable dentures in everyday life. Only a few did not wear their dentures at all or wore them sporadically (Table 4). Many of the patients were satisfied/very satisfied with their dentures; the dentures were worn with greater satisfaction in the upper jaw than in the lower jaw. About one fifth of the participants stated that they were neutral, dissatisfied or very dissatisfied with their upper dentures (lower jaw: 39.5%) (Table 4).

**Table 4:** Wearing habits and satisfaction with removable dentures in the upper jaw and lower jaw

*“Do you wear your dentures?”*	Upper jaw		Lower jaw	
	*n* = 45		*n* = 43	
Dentures are:	*n*	%	*n*	%
Worn	37	82,2	35	81,4
Worn sporadically	3	6,7	1	2,3
Not worn	4	8,9	5	11,6
Not specified	1	2,2	2	4,7
Total	45	100	43	100
*“When do you wear your prosthesis?”*	*n*	%	*n*	%
Day and night	23	51,2	22	51,2
Only during the day	15	33,3	15	34,9
Only to eat	1	2,2	1	2,3
Never	4	8,9	4	9,3
Not specified	2	4,4	1	2,3
Total	45	100	43	100
*“How satisfied are you with your prosthesis?”*	*n*	%	*n*	%
Very satisfied	17	37.8	9	20.9
Satisfied	16	35.6	16	37.3
Neutral	3	6.7	9	20.9
Rather dissatisfied	6	13.3	5	11.6
Very dissatisfied	2	4.4	3	7.0
Not specified	1	2.2	1	2.3
Total	45	100	43	100

### Quality of dentures and need for prosthetic treatment

Only a quarter of the upper and a third of the lower dentures were of very good quality according to Marxkors [15] and did not require any intervention (Table 5, A). Around 75% of the participants described the quality of their prostheses as good or fair (Table 5, B). Of maxillary dentures 80% (*n* = 36 of 45 upper dentures) (69.8% of lower dentures, *n* = 30 of 43 lower dentures) had defects (Table 5, C). Of denture wearers 38.5% were affected by denture-induced oral mucosal changes (e.g. ulceration with ill-fitting dentures). During hospitalization, many dentures of moderate quality could be restored with the help of the dentist and dental technician.

**Table 5:** A. Removable prosthetic treatment need in the upper jaw and lower jaw (e.g. repair and adjustment of the denture base). B. Retention of the dentures of the upper and lower jaw^a^. C. Type of defects^a^/^b^ (multiple answers possible)

	Removable dentures			
Location of the dentures	Upper jaw		Lower jaw	
A. Need for prosthetic treatment	*n* = 45	%	*n* = 43	%
No need	11	24.4	13	30.2
Repair (e.g. relining)	20	44.5	20	46.5
New fabrication	9	20.0	4	9.3
Not assessable	5	11.1	6	14.0
B. Denture retention^a^	*n* = 45	%	*n* = 43	%
Good	15	33.3	18	41.8
Fair	18	40.1	11	25.6
Poor	6	13.3	7	16.3
Not assessable	6	13.3	7	16.3
C. Type of deficiencies ^a^/^b^ (multiple answers)	58 defects on 36 (100%) of the 45 dentures		52 defects on 30 (100%) of the 43 dentures	
	*n*	%	*n*	%
Base insufficient	19	52.8	12	40
Replacement teeth worn down	12	33.3	8	26.7
Retention poor	8	22.2	8	26.7
Veneer chipped	5	5.6	9	23.3
Artificial denture teeth not replaced/fractured	3	8.3	1	3.3
Telescopic crown not filled after tooth extraction	2	5.6	2	6.7
Retention element missing	2	5.6	4	10.0
Vertical dimension reduced	1	2.8	1	3.3
Marginal excess	1	2.8	0	0
Denture is not available in hospital, not worn at home, therefore deficiency probable	5	13.9	7	23.3

^a^6 maxillary and 7 mandibular prostheses could not be assessed due to pain or the participant’s refusal to insert the prosthesis

^b^2 maxillary and 2 mandibular prostheses already had visible external defects

### Oral functional capacity

Only 8.1% of the participants are fully resilient (OFC: RCL 1) from the dentist’s point of view when receiving dental therapy (slightly reduced: 33.8%, greatly reduced: 44.6%, not resilient 13.5%). This means that 86.5% of the participants could be treated by a dentist, although almost half of them had not seen a dentist for more than 1 year.

## Discussion

### Study implementation

Half of the newly admitted patients during the study period agreed to participate in the study. Considering that some patients could not participate due to their degree of illness, the study represents a realistic sample of an acute geriatric study population.

### Need for prosthetic treatment

A diagnosis of oral infections should also be carried out by dentists as standard in the context of an acute inpatient admission to geriatric clinics [1]. Restrictions in chewing efficiency due to defects in the dentures (Table 5) are masked by changes in food selection. An assessment of masticatory efficacy can be arranged by the dental or medical staff in the hospital [16, 30, 31]. Denture-related oral mucosal changes and defects in dentures have also been confirmed by other studies [14, 26]. Mismatched dentures can traumatize the mucosa to the point of tumor-like changes. Analgesic medication often leads to patients being unaware of oral lesions. 76% of the missing teeth were replaced, indicating a high level of prosthetic treatment; however, as reported in other studies there were substantial deficiencies even though patients were often satisfied with their defective dentures [1].

### Utilization of dental services

For 21.5% of the participants the last visit to the dentist was more than 2 years ago, for 10.1% even more than 5 years ago (Table 3). A comparable study showed 46.3% (31.7% < 1–5 years, 14.6% > 5–25 years) [1] had not visited the dentist for more than 12 months. Reasons for a reduced acceptance of dental services can be, among others, restrictions in the state of health, lack of (mobile) dental care close to home or a low social status [24]. Protective and modifying factors in relation to the utilization of dental services by seniors can be found at different levels of the healthcare system. Static and dynamic factors influencing a reduced utilization can occur isolated or in combination and, thus, model the risk of a reduced utilization of dental services. Protective factors of utilization include patient-specific factors for self-motivation and factors that promote oral health-related resilience. Resistance forces that counteract can be identified as oral health-related resilience factors [4, 10, 20]. Therefore, inpatient acute geriatric rehabilitation could offer a goal-promoting opportunity to reintroduce patients without continuous care to dental care. Costly transport and time-consuming consultation situations between general practitioners and dentists (e.g. necessity of discontinuing anticoagulants) could be additionally avoided in this way.

### Limitations

In order to increase the significance of the study results, the following changes in the study design would be necessary:

- Varying numbers of participants in parameters examined are partly due to the multimorbidity of many participants in an inpatient hospital situation. Interviews with relatives could provide additional information on some parameters.
- A radiological examination would help to assess dental treatment needs more comprehensively.
- A larger sample could provide differentiation of subjects by cognitive ability for more in-depth assessment of the type of dental treatment required.
- To shorten or shift the content of the interview, information, e.g. on the last visit to the dentist and on the last dental treatment could also be requested from the general dentist.

## Conclusion

The study proves a need for prosthetic dental treatment in patients who are treated as inpatients in acute geriatric care. Comparable studies also showed a need for oral treatment [1, 3, 8, 21]. To promote oral health in acute geriatric patients, it is necessary for physicians to realise the importance of oral health in the context of general medical conditions [2, 8, 27, 29]. The available data also confirm that the use of validated assessment tools by physicians, such as the mini dental assessment (MDA) [16, 31] as part of the comprehensive geriatric assessment (CGA) [5, 12] would be useful. The MDA, for example, assesses chewing efficiency with the carrot-based chewing function test. The influence of good nutrition on general health is well known. Among other factors, good nutrition depends on the ability to crush food and to insalivate the bolus [6, 11, 28, 30]. Dental screening with indications for the physician on the urgency of treatment and dental treatment considering the patient’s oral functional capacity would be desirable in an acute geriatric facility. Dental screening would ideally complement the comprehensive rehabilitative approach of acute geriatric treatment [13, 25]. The information gathered could be included in the discharge report and benefit the general practitioner of the patient, with information about the oral situation and any treatment required, e.g. the presence of periodontitis. [27, 29]. The aims of oral geriatric treatment are freedom from oral pain, the reduction of oral sources of infection and the restoration of chewing function (e.g. through filling therapy and denture repairing) to facilitate and support general rehabilitation of the patient. Dental care close to home could be arranged in cases where dental treatment during in-patient acute geriatric care is not completed and for all patients to facilitate access to regular preventive care at home. The remuneration of dental services that are not located in the inpatient care service must be clarified.

### Take home message

Patients in acute geriatric care present with oral prosthetic problems.

Therefore

- an oral health assessment to diagnose oral disease and dental prosthesis defects should be included in comprehensive rehabilitation,
- conditions identified should be treated by the dentist during the inpatient rehabilitation measure and be financed by the health insurance,
- dental care for patients in acute geriatrics should be developed for a more equal access to care,
- patients should be referred to dental care close to home after being discharged from the hospital.

## Declarations

The ethics committee of the Berlin Charité approved the implementation of the study (ethics vote of 25 June 2015, application number EA 1/139/15).
